# Observation of Gonio Structures during Microhook Ab Interno Trabeculotomy Using a Novel Digital Microscope with Integrated Intraoperative Optical Coherence Tomography

**DOI:** 10.1155/2020/9024241

**Published:** 2020-07-17

**Authors:** Akiko Ishida, Kazunobu Sugihara, Tomoki Shirakami, Aika Tsutsui, Kaoru Manabe, Masaki Tanito

**Affiliations:** Department of Ophthalmology, Shimane University Faculty of Medicine, Izumo, Japan

## Abstract

**Purpose:**

Observation of ocular structures using microscope-integrated intraoperative optical coherence tomography (iOCT) has been adopted. Using the novel digital ophthalmic microscope, ARTEVO 800 with iOCT, we tested the feasibility of trabecular meshwork (TM) imaging during microhook ab interno trabeculotomy, a minimally invasive glaucoma surgery.

**Methods:**

The nasal and temporal sides of the TM/inner wall of Schlemm's canal were incised more than 3 clock hours in 14 glaucomatous eyes of 10 patients. To observe the trabeculotomy site, iOCT was performed with the real-time five-line scan mode under observation using a Swan-Jacob gonioprism lens. The success of the imaging and visibility of the trabeculotomy cleft and its incisional patterns (i.e., anterior, middle, or posterior pattern) were determined by reviewing the iOCT video files.

**Results:**

OCT images of the region of interest were acquired successfully in 100% of the 28 nasal or temporal sides in 14 eyes, although the trabeculotomy cleft was not visualized in four (14%) sides due to blockage of the OCT signal by a blood clot. Based on the predominant locations of the TM flaps in 24 of the acquired images, the trabeculotomy clefts were classified as anterior incisional patterns in 13 (54%), middle incisional patterns in nine (38%), and posterior incisional patterns in two (8%).

**Conclusion:**

Intraoperative imaging of the gonio structures including the trabeculotomy cleft was feasible using the ARTEVO 800 with iOCT in combination with a gonioprism.

## 1. Introduction

Trabeculotomy is a glaucoma surgery that reduces intraocular pressure (IOP) by eliminating aqueous flow resistance by cleavage of the trabecular meshwork (TM) and inner walls of Schlemm's canal at the point of outflow resistance of the aqueous humor. A new technique, i.e., the ab interno approach, for performing trabeculotomies has been reported recently in which the TM is incised or excised using specialized devices under direct observation of the anterior-chamber angle structure [1, 2]. We initially reported a case of both eyes of one patient with steroid-induced glaucoma who underwent a novel ab interno trabeculotomy, which we referred to as microhook ab interno trabeculotomy (*μ*LOT) [3]. Because of the substantial IOP decrease in that case, we began to perform the procedure in other cases and reported the early postoperative results and safety profile of *μ*LOT [4–7]. The features of *μ*LOT, which include conjunctival and scleral sparing with the ab interno technique, short surgical time, moderate IOP reduction, and no bleb-related complications, fulfill the conditions of minimally invasive glaucoma surgery (MIGS) [8].

Optical coherence tomography (OCT) is an indispensable diagnostic tool for managing numerous ophthalmic diseases. Observation of the ocular microarchitectural structures using microscope-integrated intraoperative OCT (iOCT) was initially conducted using an external portable OCT system mounted on a microscope [9] and, more recently, using an OCT system integrated into surgical microscopes [10]. iOCT was adopted initially in vitreoretinal surgery to assess macular holes and epiretinal membranes [10], in corneal surgery to visualize the donor cornea during endothelial keratoplasty [11], and in cataract surgery to evaluate the intraocular lens position [12]. Several studies have used the iOCT device during glaucoma surgeries to image the scleral lake during canaloplasty [13], refine bleb needling [14], assess gonio structures during Trabectome (NeoMedix, Tustin, CA, USA) procedures [15], adjust the tube position during tube-shunt surgery [16], and assess changes in the angle recess in plateau iris syndrome [17].

We previously reported the feasibility results of gonio structure observation during MIGS using a microscope-iOCT device (RESCAN 700, Carl Zeiss Meditec AG, Jena, Germany) [18]. In the current study, we report our initial feasibility assessment using the novel digital ophthalmic microscope (ARTEVO 800 with iOCT, Carl Zeiss Meditec AG) to visualize the incised TM observation during *μ*LOT.

## 2. Methods

This retrospective observational study included 14 consecutive glaucomatous eyes of 10 subjects (5 men, 5 women; mean ± standard deviation age, 66.9 ± 9.5 years, range 47–78 years) who underwent *μ*LOT with iOCT to reduce IOP in February 2020. The study adhered to the tenets of the Declaration of Helsinki; the institutional review board (IRB) of Shimane University Hospital reviewed and approved the research (No. 20200227-2). Preoperatively, all subjects provided written informed consent for surgery; however, the IRB approval did not require that each patient provide written informed consent for publication; instead, the study protocol was posted at the study institutions to notify participants about the study. The subjects' demographic data included the glaucoma types, i.e., six (43%) eyes with primary open-angle glaucoma, five (36%) with pseudoexfoliation glaucoma, two (14%) with mixed-mechanism glaucoma, and one (7%) with steroid-induced glaucoma. Eleven (79%) eyes were phakic and three (21%) were pseudophakic. No eye had a history of having undergone a previous glaucoma surgery. The procedure included *μ*LOT alone in six (43%) eyes and *μ*LOT combined with cataract surgery in eight (57%) eyes.

**Figure 1:**
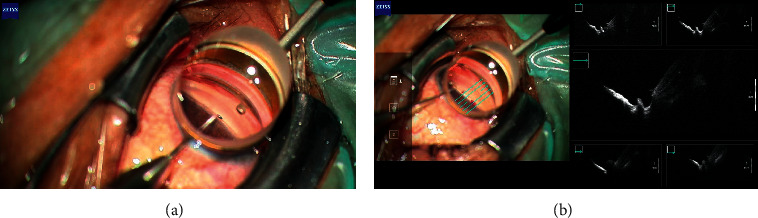
Intraoperative integrated optical coherence tomography (iOCT) during microhook ab interno trabeculotomy. (a) Intraoperative findings during microhook ab interno trabeculotomy. Under observation using a Swan-Jacob gonioprism lens, a microhook is inserted into Schlemm's canal. In this left eye, the nasal angle is being incised with the straight microhook that is inserted from the temporal corneal port. (b) Intraoperative observation of the incised trabecular meshwork and inner wall of Schlemm's canal by five-line scans using the ARTEVO 800 with iOCT in combination with a Swan-Jacob gonioprism lens. In this left eye, the nasal angle is visualized with iOCT. The green arrows indicate the scan direction.

The *μ*LOT procedure was performed through two corneal side ports as reported previously [1]. Briefly, a spatula-shaped microhook (M-2215, Inami, Tokyo, Japan) was used, which was designed specifically for use during *μ*LOT. Viscoelastic material (1% sodium hyaluronate, Healon, AMO Japan, Tokyo, Japan) was injected into the anterior chamber through the clear corneal ports created using a 20-gauge micro-vitreoretinal knife (Mani, Utsunomiya, Japan) at the 2–3 and 9–10 o'clock positions. A microhook was inserted into the anterior chamber through the corneal port using a Swan-Jacob gonioprism lens (Ocular Instruments, Bellevue, WA, USA) to observe the angle opposite to the corneal port. The tip of the microhook then was inserted into Schlemm's canal and moved circumferentially to incise the inner wall of Schlemm's canal and TM over 3 clock hours (Figure 1(a)). Using the same procedure, LOT was performed in the opposite angle using a microhook inserted through the other corneal port. In cases of combined surgery, before *μ*LOT, phacoemulsification cataract surgery was performed through a 2.2 mm wide clear corneal incision created at the 9–10 o'clock position (i.e., temporal incision for the right eye and nasal incision for the left eye); a one-piece soft acrylic intraocular lens (Vivinex iSert XY1, Hoya, Tokyo, Japan) was inserted through the same clear corneal incision. Then, *μ*LOT was performed through the 2.2 mm clear corneal incision and a clear corneal port created at the 2–3o'clock position in both eyes.

iOCT then was performed to assess the gonio structures in the temporal and nasal sides of each eye, i.e., a total of 28 images from the 14 eyes, using the ARTEVO 800 with iOCT that was equipped with spectral domain OCT. Because of poor penetration of the light source, the angle structure was not visualized through the limbal tissue. Alternatively, the gonio structure was visualized under observation with a Swan-Jacob gonioprism lens using the real-time five-line scan mode (scan width, 6.0 mm; scan interval, 0.75 mm; scan depth 2.9 mm) (Figure 1(b), Video 1). The iOCT image was recorded in mp4 format. After iOCT imaging, the viscoelastic material was aspirated through the handpieces and the corneal ports were closed by corneal stromal hydration. Typically, surgical time of combined and solo surgeries were 10 and 4 minutes, respectively; and additional 2 minutes were required to obtain iOCT images in 2 locations (i.e., 1 minute each for nasal or temporal angle).

**Figure 2:**
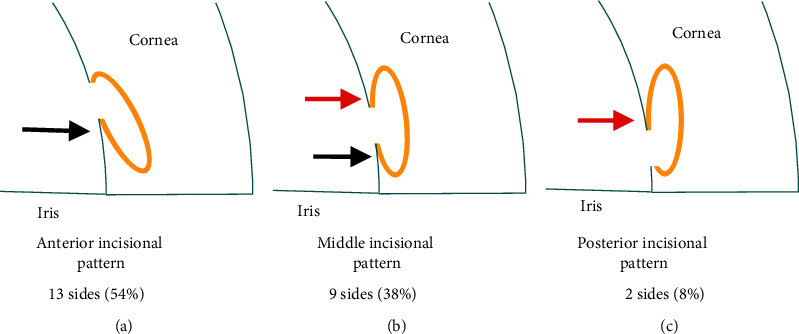
Schematic drawings of the three patterns of the trabeculotomy clefts. Based on the flap locations (black and red arrows), the trabeculotomy cleft is classified into an anterior (a), middle (b), or posterior (c) incisional patterns. The black and red arrows indicate posterior- and anterior-based flaps, respectively. The yellow ovals indicate Schlemm's canal. This figure is a modification of our previous publication in the *Journal of Ophthalmology* [18].

One author (MT) reviewed the iOCT videos and assessed the success of imaging, visibility of the trabeculotomy cleft, and incisional patterns (i.e., anterior, middle, or posterior pattern) (Figure 2) according to a previous report on the RESCAN 700 [18].

## 3. Results

At the final follow-up visit at 1.6 ± 0.6 months postoperatively, the baseline IOP of 20.4 ± 3.3 mmHg decreased to 15.0 ± 3.9 mmHg (25% reduction, *P* < 0.0001, Wilcoxon signed-rank test). The baseline number of glaucoma medications of 3.1 ± 1.3 remained unchanged at 2.7 ± 0.6 (*P*=0.1386). The baseline best-corrected visual acuity of 0.2 ± 0.4 in logarithm of the minimum angle of resolution (logMAR) notation was unchanged at 0.1 ± 0.3 (*P*=0.6108), and the visual acuity did not decrease in any eye more than 0.2 logMAR. Other than perioperative hyphema in all eyes and a transient IOP elevation of more than 30 mmHg in two (14%) eyes, no surgery-related complications were recorded.

**Figure 3:**
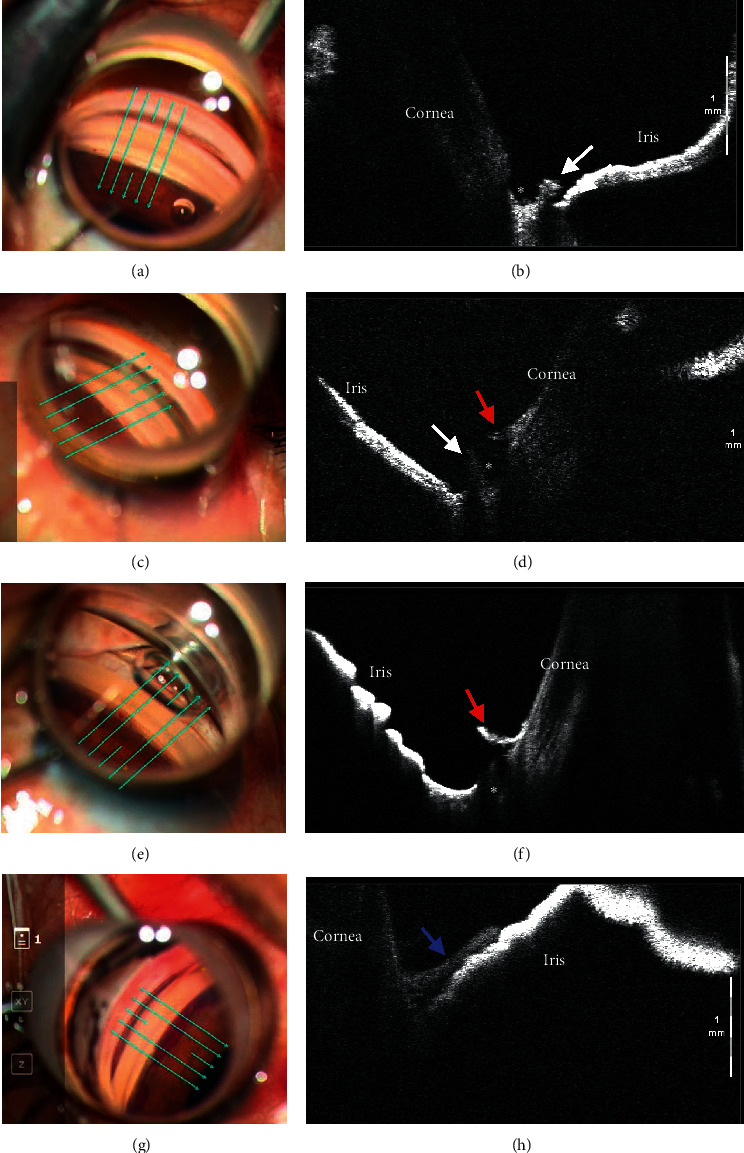
Representative microscope-integrated optical coherence tomography (OCT) images of trabeculotomy site. Based on the flap locations (white and red arrows), the trabeculotomy cleft are classified into an anterior incisional pattern (a, b) (seen with posterior-based flaps predominantly), middle incisional pattern (c, d) (seen with posterior- and anterior-based flaps), or posterior incisional pattern (e, f) (seen with anterior-based flap predominantly). (g, h) The trabeculotomy cleft is unclear because the OCT signal is blocked by a blood clot. The white arrows indicate a posterior-based flap; red arrows, an anterior-based flap; blue arrow, a blood clot; green arrows, the direction of the scan; and asterisk, the lumen of Schlemm's canal.

A review of the surgical videos showed that the OCT images successfully captured the areas of interest in all (100%) of the 28 nasal and temporal angles in 14 eyes in which iOCT was performed. Of them, the trabeculotomy cleft was seen in 24 (86%) nasal and temporal sectors of 14 eyes (Figures 3(a)–3(c)) but was not visualized due to blockage of the OCT signal in four (14%) sides in four eyes (Figure 3(d), blue arrow). Of the 24 sides, based on the appearance of the acquired images, the incisional patterns were classified as anterior incisional patterns in 13 (54%) (Figure 3(a), posterior-based flap), middle incisional patterns in nine (38%) (Figure 3(b), posterior- and anterior-based flaps), and posterior incisional patterns in two (8%) (Figure 3(c), anterior-based flap), according to the predominant locations of the TM flaps.

## 4. Discussion

Using the RESCAN 700, Siebelmann et al. reported that good visualization of the gonio structures can be achieved through a deep sclerectomy window during canaloplasty [13]; while Junker et al. [15] and we [18] reported that Schlemm's canal could not be visualized through the full-thickness sclera because of poor penetration of the 840 nm light source. In the current study, we reconfirmed that Schlemm's canal could not be visualized through the full-thickness sclera using the ARTEVO 800. In contrast to direct observation, we successfully observed a trabeculotomy cleft and the lumen of Schlemm's canal using the combination of the ARTEVO 800 and a gonioprism. Previously, using the RESCAN 700 and a gonioprism, OCT images were not obtained in 17% of observations due to the lengthy time required to frame/focus the image [18]. Junker et al. pointed out that one of the biggest problems associated with gonio-structure imaging by iOCT is focusing on the region of interest [15]; thus, iOCT of the gonio structures performed in combination with a gonioprism requires experience for successful image acquisition. In the current study, OCT images were obtained for 100% of eyes using iOCT. In addition to the improved skills of the operator/surgeons, improvement in hardware/operating software (e.g., ease of focusing) of the ARTEVO 800 compared with the RESCAN 700 might be associated with this increased image acquisition, but this is inconclusive since the detailed specifications of the iOCT device of the ARTEVO 800 have not been released. Real-time iOCT “during” the TM incision, rather than “after” the TM incision, is ideal, although the “during” iOCT is still difficult to perform with current ARTEVO800; thus, further improvement of its usability is desired.

According to our previous study, based on the appearance of the acquired images of the 24 sides, we successfully classified the trabeculotomy cleft into three incisional patterns. In our previous study using the RESCAN 700, the patterns were determined as comprising 60%, 30%, and 10% of the anterior, middle, and posterior incisional patterns, respectively, during *μ*LOT [18]. Therefore, we confirmed that the cleft at the anterior edge of the TM was the most frequently observed pattern after the incision using a microhook; this should be associated with significant IOP reductions after *μ*LOT, although the association between the patterns or extent of TM incision and surgical efficacy needs clarification.

In conclusion, intraoperative imaging of the gonio structures including the trabeculotomy cleft was feasible using the ARTEVO 800 with iOCT in combination with a gonioprism. This technique might be useful to confirm the proper incision of the inner wall of Schlemm's canal during trabeculotomy.

## Data Availability

The data used to support the findings of this study are included within the article.

## Supplementary Materials

Supplementary Materials Video 1: real-time observation of the incised trabecular meshwork using the ARTEVO 800 with microscope-integrated optical coherence tomography in combination with a gonioprism.

## References

[1] Tanito M. (2018). Microhook ab interno trabeculotomy, a novel minimally invasive glaucoma surgery. *Clinical Ophthalmology*.

[2] Tanito M., Matsuo M. (2019). Ab-interno trabeculotomy-related glaucoma surgeries. *Taiwan Journal of Ophthalmology*.

[3] Tanito M., Sano I., Ikeda Y., Fujihara E. (2016). Microhook ab interno trabeculotomy, a novel minimally invasive glaucoma surgery, in eyes with open-angle glaucoma with scleral thinning. *Acta Ophthalmologica*.

[4] Tanito M., Sano I., Ikeda Y., Fujihara E. (2017). Short-term results of microhook ab interno trabeculotomy, a novel minimally invasive glaucoma surgery in Japanese eyes: initial case series. *Acta Ophthalmologica*.

[5] Tanito M., Ikeda Y., Fujihara E. (2017). Effectiveness and safety of combined cataract surgery and microhook ab interno trabeculotomy in Japanese eyes with glaucoma: report of an initial case series. *Japanese Journal of Ophthalmology*.

[6] Tanito M., Matsuzaki Y., Ikeda Y., Fujihara E. (2017). Comparison of surgically induced astigmatism following different glaucoma operations. *Clinical Ophthalmology*.

[7] Tanito M., Manabe K., Mochiji M., Takai Y., Matsuoka Y. (2019). Comparison of anterior chamber flare among different glaucoma surgeries. *Clinical Ophthalmology*.

[8] Ahmed I. I. K (2015). MIGS and the FDA: what’s in a name?. *Ophthalmology*.

[9] Ehlers J. P., Dupps W. J., Kaiser P. K. (2014). The Prospective Intraoperative and Perioperative Ophthalmic ImagiNg With Optical CoherEncE TomogRaphy (PIONEER) study: 2-year results. *American Journal of Ophthalmology*.

[10] Ehlers J. P., Modi Y. S., Pecen P. E. (2018). The DISCOVER study 3-year results. *Ophthalmology*.

[11] Kobayashi A., Yokogawa H., Mori N., Sugiyama K. (2016). Visualization of precut DSAEK and pre–stripped DMEK donor corneas by intraoperative optical coherence tomography using the RESCAN 700. *BMC Ophthalmology*.

[12] Lytvynchuk L. M., Glittenberg C. G., Falkner-Radler C. I. (2016). Evaluation of intraocular lens position during phacoemulsification using intraoperative spectral-domain optical coherence tomography. *Journal of Cataract & Refractive Surgery*.

[13] Siebelmann S., Cursiefen C., Lappas A., Dietlein T. (2016). Intraoperative optical coherence tomography enables noncontact imaging during canaloplasty. *Journal of Glaucoma*.

[14] Dada T., Angmo D., Midha N., Sidhu T. (2016). Intraoperative optical coherence tomography guided bleb needling. *Journal of Ophthalmic and Vision Research*.

[15] Junker B., Jordan J., Framme C., Pielen A. (2017). Intraoperative optical coherence tomography and ab interno trabecular meshwork surgery with the Trabectome. *Clinical Ophthalmology*.

[16] Swaminathan S. S., Chang T. C. (2017). Use of intraoperative optical coherence tomography for tube positioning in glaucoma surgery. *JAMA Ophthalmology*.

[17] Pathak Ray V., Puri V., Peguda H. K., Rao D. P. (2019). Intra-operative ASOCT determined changes in angle recess in plateau iris syndrome post phaco alone and post phaco-endocycloplasty. *Graefe’s Archive for Clinical and Experimental Ophthalmology*.

[18] Tanito M. (2017). Optical coherence tomography observation of gonio structures during microhook ab interno trabeculotomy. *Journal of Ophthalmology*.

